# Keyhole lesion caused by a fatal self inflicted headshot with a modified flare gun

**DOI:** 10.1007/s12024-025-01102-8

**Published:** 2025-10-07

**Authors:** Balázs Varga, Panna Jámbor-Hegedüs, Zsófia Almádi, János Bokor

**Affiliations:** 1 Department of Forensic Medicine, Hungarian Institute for Forensic Sciences, Budapest, Hungary; 3 Department of Pathology, Forensic and Insurance Medicine, Semmelweis University, Budapest, Hungary; 2 Division of Pathology and Oncology, Doctoral College of Semmelweis University, Budapest, Hungary

**Keywords:** Forensic pathology, Gunshot wounds, Tangential gunshot, Skull fractures, Keyhole lesion

## Abstract

In the following case report, we discuss the scene examination and autopsy results of an 86-year-old man who committed suicide in his closed garage by shooting himself in the head with a modified flare gun while seated in the passenger seat of his car. During the external examination of the autopsy an atypical injury on the scalp and skull showed the characteristics of both an entry and exit wound simultaneously, consistent with the phenomenon of a keyhole fracture. There are differing forensic interpretations regarding the mechanism of this fracture. One possible explanation is that it forms because of a tangential gunshot. In such cases, the kinetic energy of the bullet is transmitted to the bone surface at an oblique angle, combined with the pressure wave simultaneously affecting the calvaria and intracranial space, and secondary projectiles (bone fragments) contribute to the characteristics of this special injury as well. Other authors state that a keyhole fracture can also occur when the bullet strikes the bone perpendicularly. In our case, by comparing the findings from the scene investigation and the autopsy, it was determined that the keyhole fracture was a result of a tangentially directed shot. Moreover, considering the suicidal intent, the injury was found at an atypical location – the occipital part of the skull. Given the highly atypical injury location, injury pattern and firearm, we hope that the present case study will be of assistance to colleagues in the forensic evaluation of similar atypical gunshot injuries.

## Introduction

Gunshot injuries and gunshot related deaths are uncommon in Hungary due to the strict firearm regulations, which are even more stern since the school shooting in the University of Pécs Medical School building in 2009 [1, 2]. When a firearm related death occurs, the on-call physician or (if available) a forensic medical expert must do the external examination on the scene to decide the manner of death, while the forensic pathologist is responsible for providing a detailed assessment of the case after performing a medico-legal autopsy. In gunshot injury cases, this includes statements regarding whether the injury was potentially self-inflicted or not, the possible shooting distance, and the bullet trajectory — all of which can significantly influence direction of the investigation going forward [3, 4]. According to the available literature, keyhole fractures can be created by shallow angle shots, where the entrance wound is caused by the bullets punch out force and a bullet part shears off and travels beneath the scalp before either coming to rest or exiting [5]. A keyhole wound can be seen in such tangential, relatively superficial gunshots as well, where the exit wound is located very close to, but separate from the entry wound, or as in our case, the entry and exit wounds can be seen within one injury. These injuries simultaneously display characteristics of both entry and exit points. They are named after their distinctive keyhole shape, in which the round part represents the entry wound with its inward beveling, and the elongated part shows exit wound characteristics with outward beveling [6–8]. This injury pattern has also been described in long bone gunshot wounds as well. Recent publications suggest that keyhole fractures may also occur from gunshots that strike the surface perpendicularly [9]. Our case study presents a highly unusual manifestation of a keyhole fracture, where a self-inflicted tangential gunshot wound of the occipital region resulted in this atypical injury pattern. The evaluation of this injury, both at the scene and during the autopsy, posed a significant challenge for the forensic experts involved.

## Case report

### Scene

At dawn an 86-year-old man shot himself fatally in the head with a modified flare gun while sitting in his car in his own garage.

**Fig. 1:**
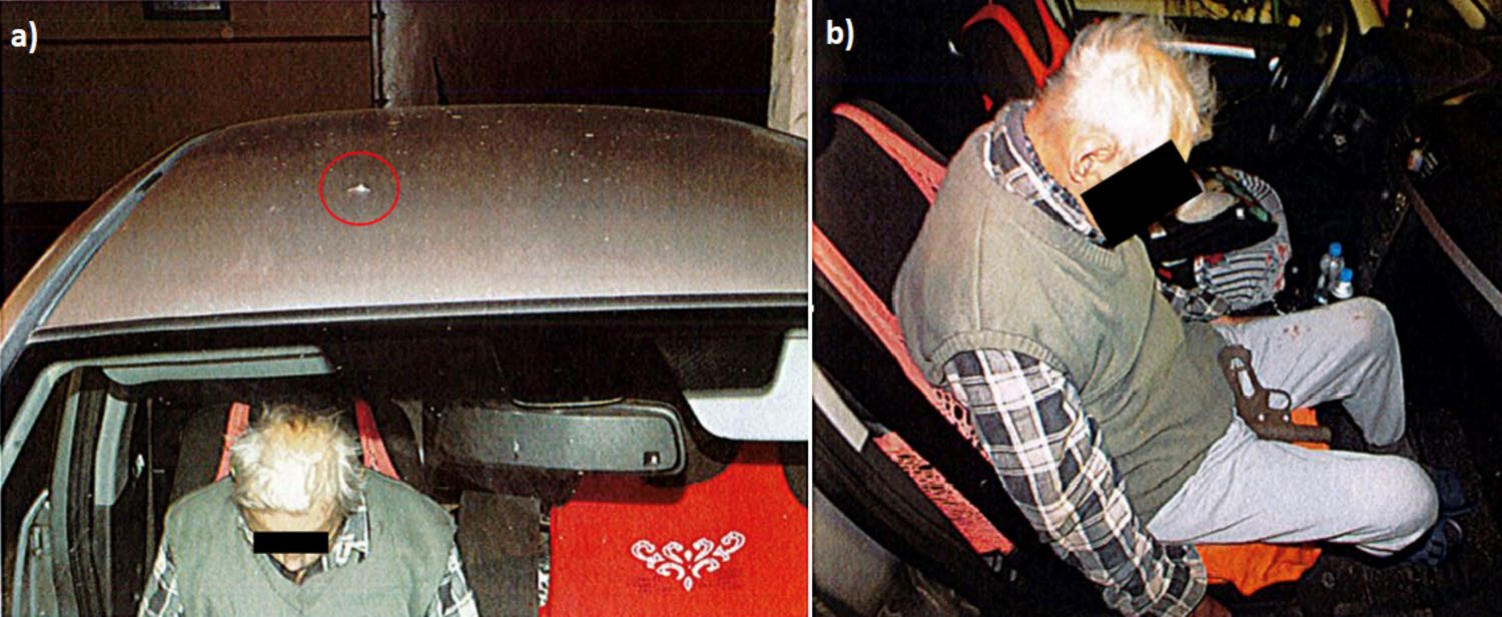
**a**) The deceased person in the position the police found him in. The roof of the car is deformed due to the gunshot (red circle marking). **b**) The deceased in the passenger seat with the flare gun between his legs and blood stain pattern on his left thigh

The police conducted a scene investigation accompanied by a forensic firearm expert and a local general physician. According to the police the deceased was found seated in the passenger seat, his head was tilted forward, and a rusted 26 mm caliber flare gun was located between his legs (Fig. 1**.**).

### Examination of the firearm and the car

**Fig. 2:**
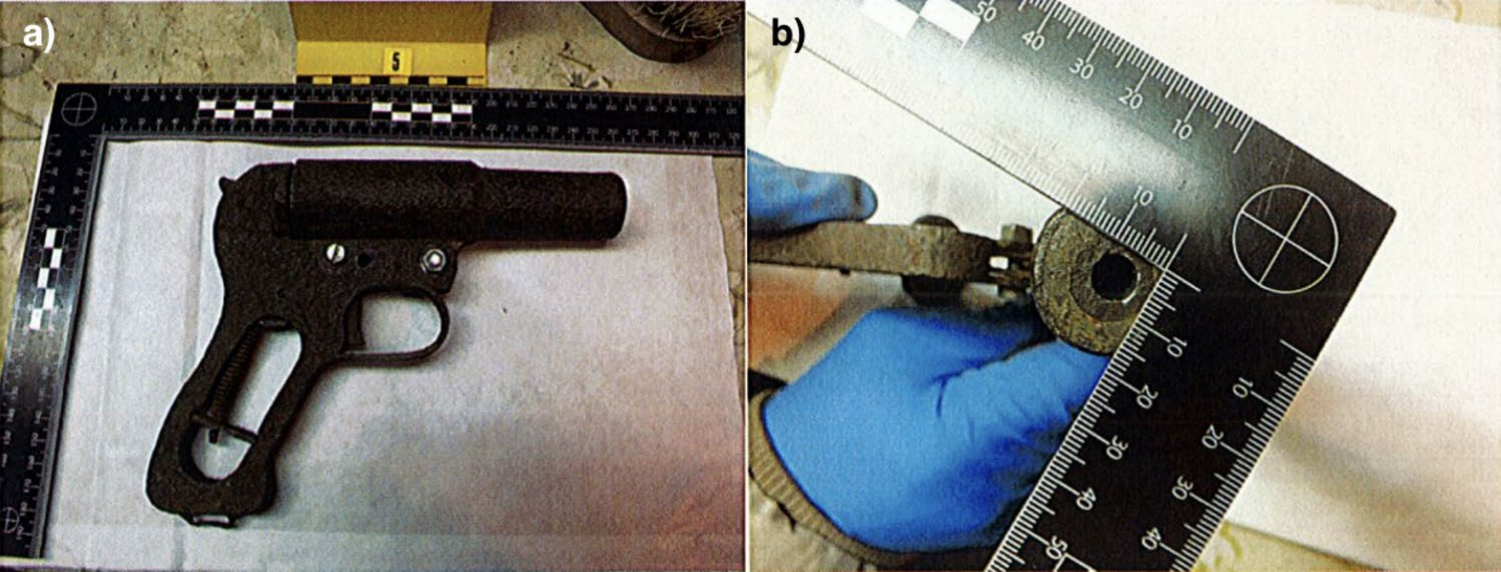
**a**) Metric photograph of the modified flare gun. **b**) Measurement of the caliber performed by the forensic firearms expert

**Fig. 3:**
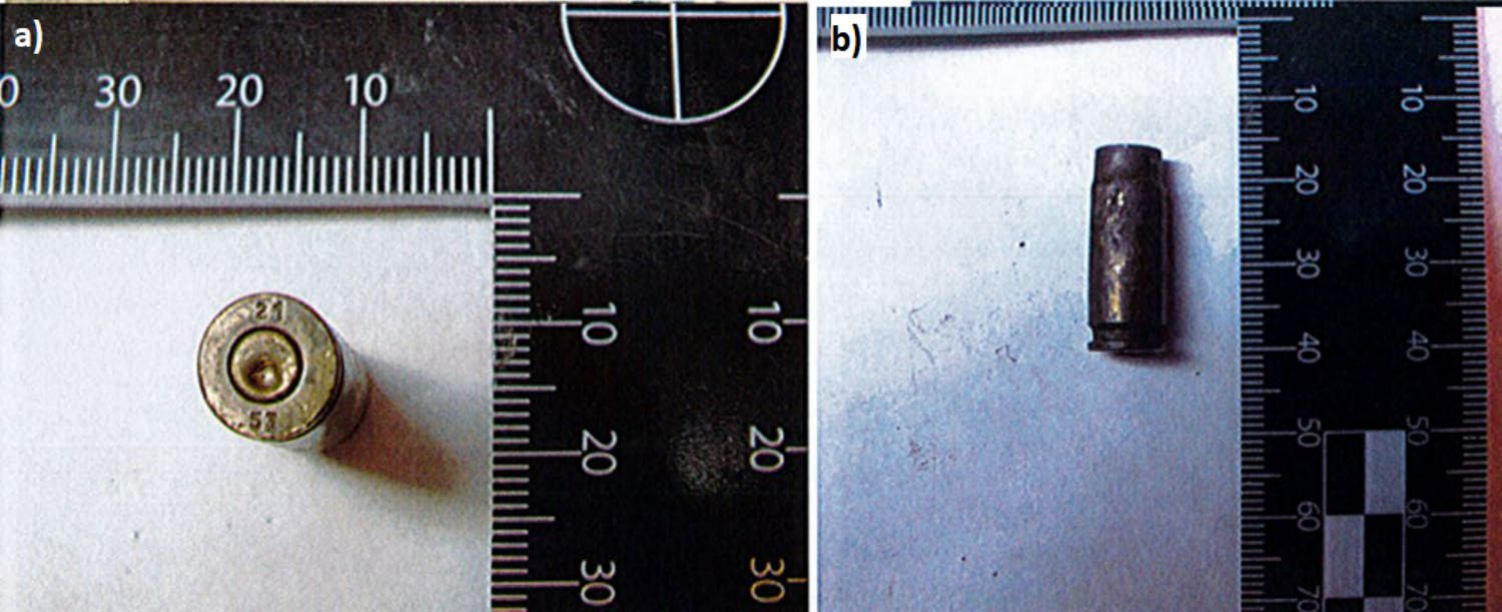
**a**) and **b**) The above represented 7.62 mm Tokarev cartridge was examined by the firearm expert. The stamp on the rear end “2153” identifies it as a Hungarian made cartridge produced in 1953. The case material was brass alloy. On the rear end a nearly circular firing pin impression was visible. The cartridge case did not contain any propellant or functional primer

The forensic firearms expert examined and disassembled the weapon. The type and serial number could not be determined. A single 7.62 mm Tokarev pistol cartridge case was removed from the gun, and DNA residue was collected from the muzzle [10] (Fig. 2–3).

The forensic firearms expert determined that the home-modified flare gun was of unknown origin. Its metal parts were heavily oxidized and rusty. A secondary barrel was added to the gun with an internal diameter of 8 mm and with an external diameter of 22 mm, which have been inserted into the original 26.5 mm bore (32 mm at the cartridge chamber). The space between the two barrels had been filled with lead.

The rear end of the inserted barrel (toward the cartridge chamber) was modified, allowing insertion of a 7.62 mm Tokarev caliber cartridge. The flare gun was capable of discharging shots, and the energy of projectiles fired from the muzzle was sufficient to cause lethal injury. Residue characteristic of firearm discharge was found in the bore, confirming that the weapon had been discharged at some point prior to examination. Successful test shots were carried out using 7.62 mm Tokarev caliber cartridges. The confiscated 7.62 mm Tokarev pistol/submachine gun cartridges were compatible with the modified flare gun. The weapon met the Hungarian safety standards—it could not be fired without pulling the trigger.

During the examination of the car, a 15 × 8 mm irregularly shaped material discontinuity in the roof of the vehicle was discovered, which was caused by a gunshot. This was removed for further investigation by the Crime Scene Technicians. A deformed alloy bullet fragment, weighing 0.3 g and measuring 6 × 6 × 2.5 mm was found embedded in the insulation.

### External body examination at the scene

During the scene examination while examining the body, the on-call physician found a lesion in the left occipital region, which he interpreted as a typical gunshot exit wound. No statements were made about the entrance wound. According to the police, the relatives revealed that the deceased had recently been complaining about pain due to an abscess on his hip, and two weeks prior to the incident, he had been making remarks such as “*why am I even alive*?!”. On the day of the incident, a gunshot or some similarly loud noise was heard. During the scene examination, no evidence of foul play was uncovered, therefore, based on the findings of the scene investigation, the case was determined by authorities as a suicide.

### Autopsy

**Fig. 4:**
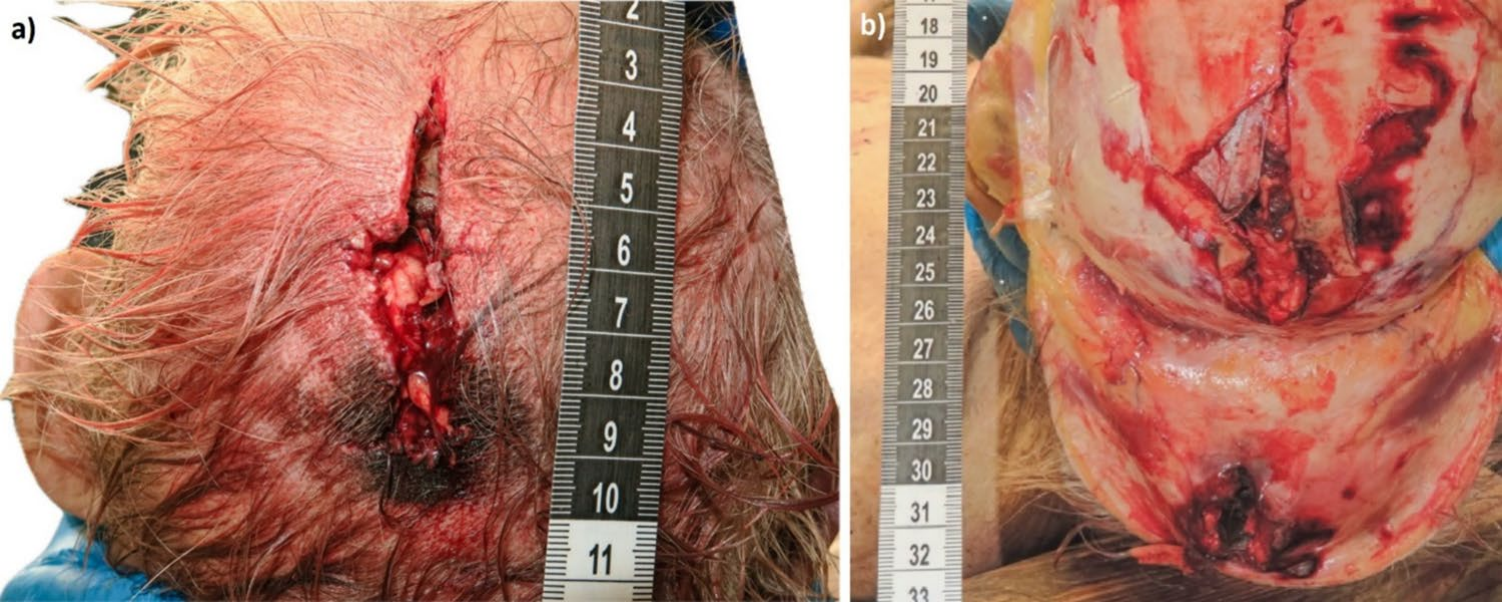
**a**) The left occipital region of the head, next to the midline. The wound contains both entrance and exit wound characteristics. At the lower part of the injury, soot particles embedded in the skin surface can be seen. **b**) On the inner surface of the scalp, hemorrhages can be observed. In the projection of the keyhole fracture, the disrupted dura mater and the injured brain tissue are exposed. Additional linear fractures radiate from the original fracture

During dissection, on the skin covering the occipital bone, a 6.5 cm long, 1.5 cm wide, irregular injury was observed. At the base of the wound, fractures of the cranial vault and brain matter were visible (Fig. 4**.**).

In the lower rim of the wound, a U-shaped, black soot-stained discoloration and cherry reddish blood staining was visible. No other external injuries were observed. The internal examination of the scalp and calvaria revealed a haematoma, and soot deposits.

**Fig. 5:**
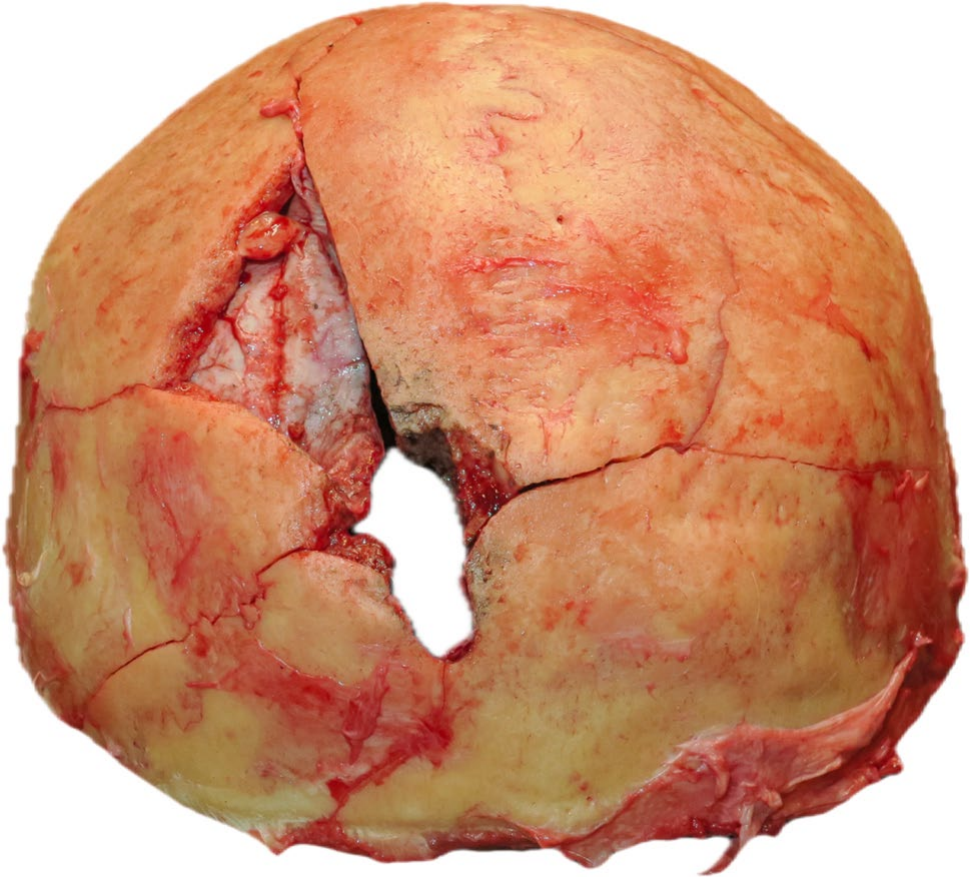
The calvaria with the so-called keyhole fracture

**Fig. 6:**
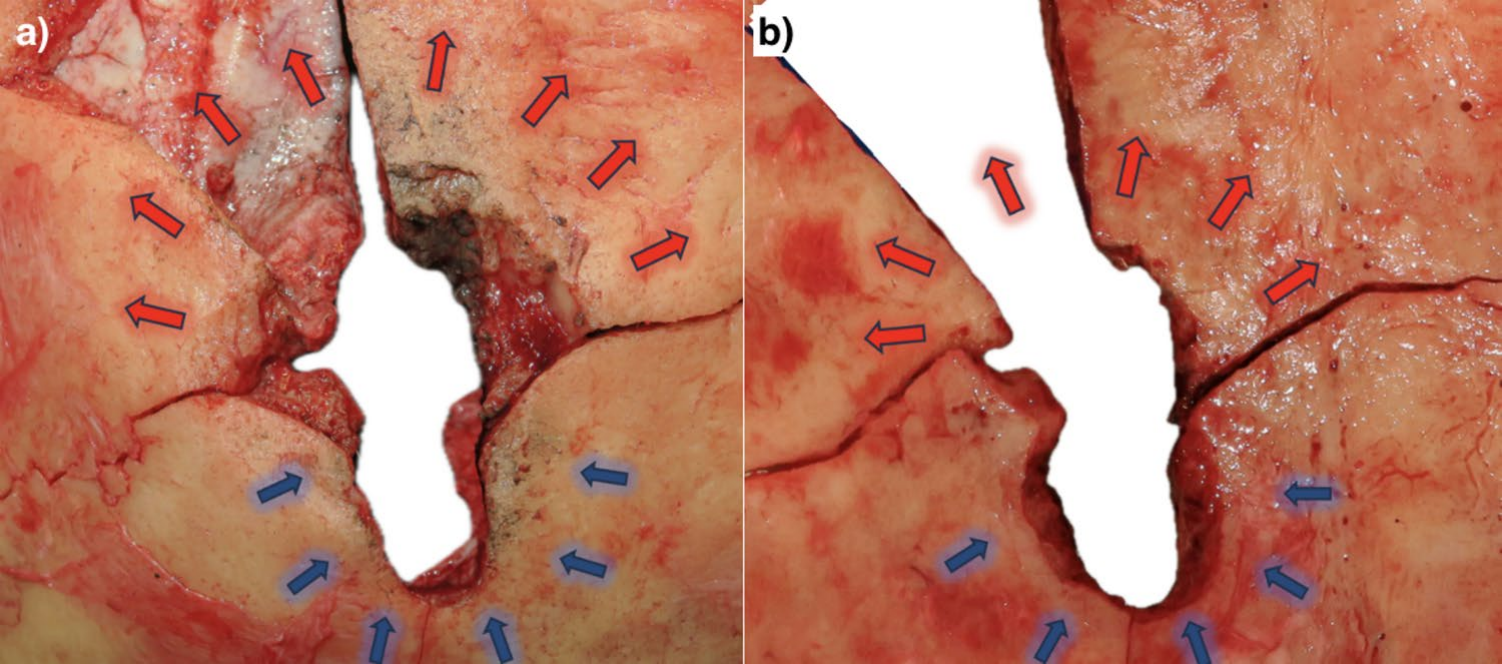
The keyhole lesion from close up. **a**) Outward beveling is shown from the external surface. **b**) Invard beveling is shown from the internal surface. Red arrows indicate exit, and blue arrows indicate entrance wound characteristics

Beneath this external injury, on the left side of the occipital bone, a downward-facing, slightly left-tilted "keyhole"-shaped fracture was found measuring about 8 × 5 cm (Fig. 5**.**). Internal beveling was found in the lower third with embedded soot particles, and external beveling was seen along the rim in the upper third. Linear fractures radiated outward from the keyhole fracture **(**Fig. 6**.)**. At the base of the injury, on the dura mater there was a 3 × 2 cm irregular tear with ragged edges and brain tissue were exposed.

On the posterior brain surface and on the left parietal and temporal lobes, fingertip-sized subarachnoid hemorrhages were found. Beneath these areas pinpoint hemorrhages were visible in the grey matter. In the cerebral ventricles blood-stained cerebrospinal fluid was also found.

On the surface of the cerebellar tonsils, numerous small (1–2 mm), sharp bone fragments were embedded into the brain matter [11–13].

The skull base and the other internal organs were intact and nothing remarkable was found, toxicological analysis was also negative.

## Discussion

In summary, related to the gunshot wound, we found scalp hemorrhage, multiple linear fractures of the cranial vault, a so-called keyhole fracture of the skull, extensive subarachnoid hemorrhages, brain contusion, secondary intracerebral hemorrhages, and secondary projectiles (bone fragments) embedded into the posterior brain surface.

Toxicological analysis confirmed that the deceased was not under the influence of alcohol, and no signs of poisoning or drug use could be forensically verified.

Both the entry and exit wounds were located in the occipital region, forming a keyhole fracture, which was caused by a tangential gunshot [14]. In this case, the kinetic energy originated by the projectile striking the occipital area at an oblique angle, along with the resulting secondary pressure waves (vertical and horizontal forces) on the calvaria and cranial cavity, as well as secondary projectiles (bone fragments), caused the documented skull fractures and brain injuries.

The lower third of the external wound was determined to be the entry site; the projectile traveled upward, slightly forward and to the left, exiting at the upper third of the wound. This trajectory is consistent with prior cases described in international literature [15].

The presence of a soot-stained discoloration at the entry site, the pattern of skull fractures, the diffuse brain damage, the autopsy findings and the police report all support the conclusion that the injury most likely was a result of a single close-ranged gunshot. Although the shot location was highly unusual, the injury could still have been self-inflicted [16]. Based on all findings—police report, medical history, toxicology results, and autopsy—we concluded that the individual’s death occurred due to diffuse brain injury caused by a gunshot to the head.

Despite the unusual location and morphology of the injury, its mechanism could be reconstructed. The detailed information regarding the position of the deceased and the modified firearm, along with the absence of any signs of foul play, provided sufficient evidence to support the conclusion of a suicide.

## Conclusion

As mentioned above, the so-called keyhole fractures are rare, their clinical and forensic relevance makes their recognition and understanding essential. For the primary care provider (such as emergency doctors) identifiying this injury is important for correctly assessing its severity. This can be crucial given the frequent discrepancy between the external wound and the extent of the intracranial injury, especially by the bone fragments as secondary projectiles within the cranial cavity.

The forensic medical expert can use the distinct characteristics of a keyhole fracture to draw appropriate conclusions about the mechanism of injury, including the location of entry and exit wounds, the shooting distance, direction of fire, and the extent of the bullet’s path in the body. When necessary, the involvement of a firearms expert during the scene investigation, and post-mortem examination, can assist in answering further questions.

## Key points

1. A tangential gunshot can produce a keyhole-shaped injury on the skull that shows both entry and exit wound characteristics.
2. The unusual placement of a gunshot wound or the use of an atypical firearm can mislead even an experienced investigator.
3. Differentiating between entry-type and exit-type beveling is crucial for determining the direction and angle of the shot.
4. Consultation with a forensic firearms expert is essential for accurately reconstructing the shooting.
